# Supplementary material for: Nanoarchitecture and dynamics of the mouse enteric glycocalyx examined by freeze-etching electron tomography and intravital microscopy
Source: Commun Biol. 2020 Jan 7;3:5. doi: 10.1038/s42003-019-0735-5 (PMC6946683; doi:10.1038/s42003-019-0735-5)
Supplement: Supplementary file 1 — Supplementary Information [file 42003_2019_735_MOESM1_ESM.pdf]

Supplementary Figure 1

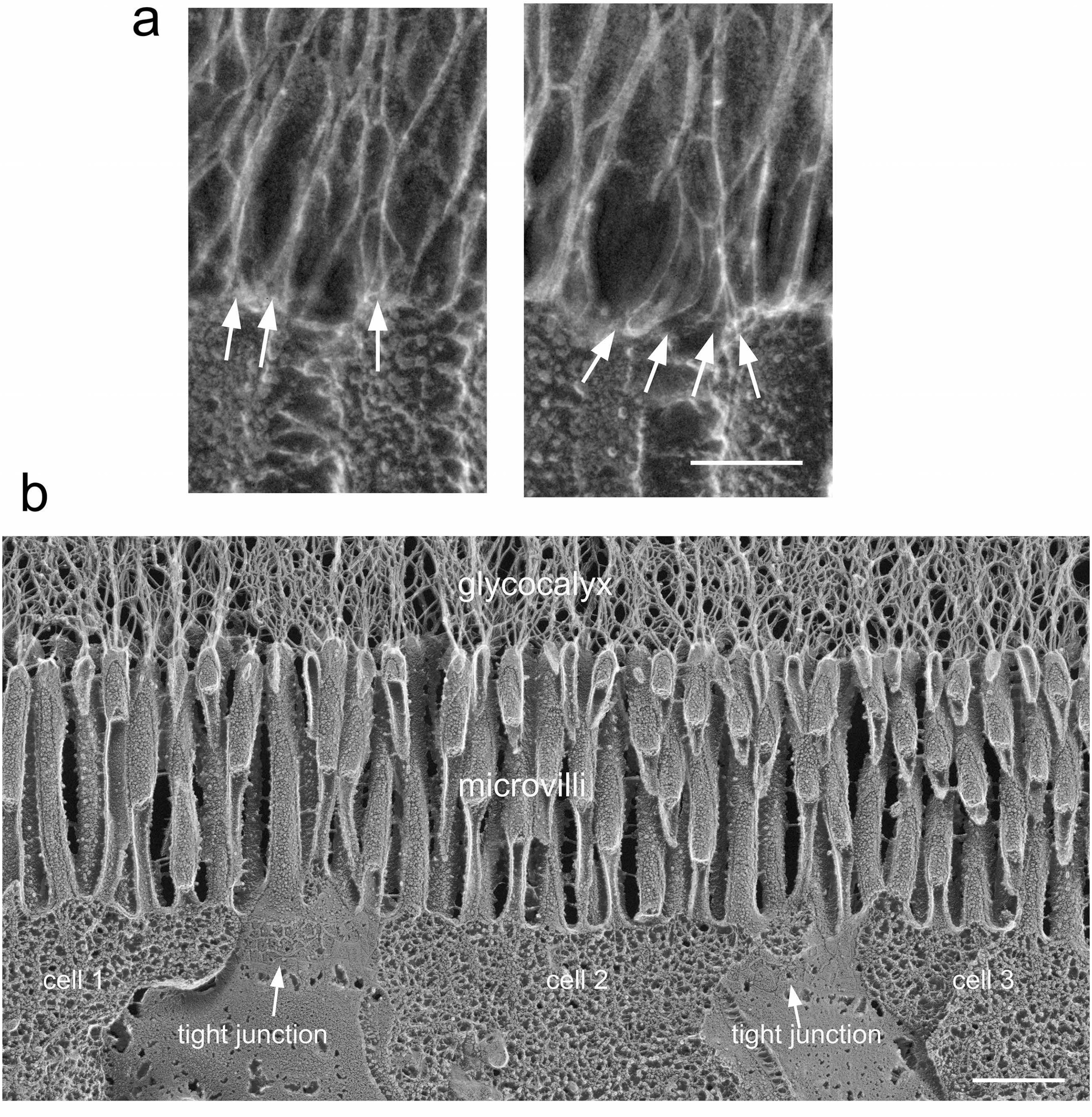

Supplementary Information

Supplementary Figure 1: The enteric glycocalyx is a transcellular network formed by interwoven filaments that emerge from the microvilli tips. **(a)** Close up view of the interface between the microvilli and the glycocalyx layer shows that multiple glycocalyx filaments emerge specifically from the distal ends, or tips, of individual microvillus and form lateral interactions, including with filaments emerging from adjacent microvilli (arrows). While most filaments appear to coalesce to form the micron long glycocalyx network, we cannot exclude that some of these filaments correspond to shorter transmembrane mucins. **(b**) Freeze-etching across multiple enterocytes (indicated) shows that the overlaying glycocalyx layer traverses cell boundaries without organizational changes between cells to form a transcellular continuum. The boundaries of the enterocytes can be clearly delineated by the presence of both P- and E-face tight junction fibrils (arrows). Scale bars: **(a)** 100 nm; **(b)** 500 nm

Supplementary Figure 2

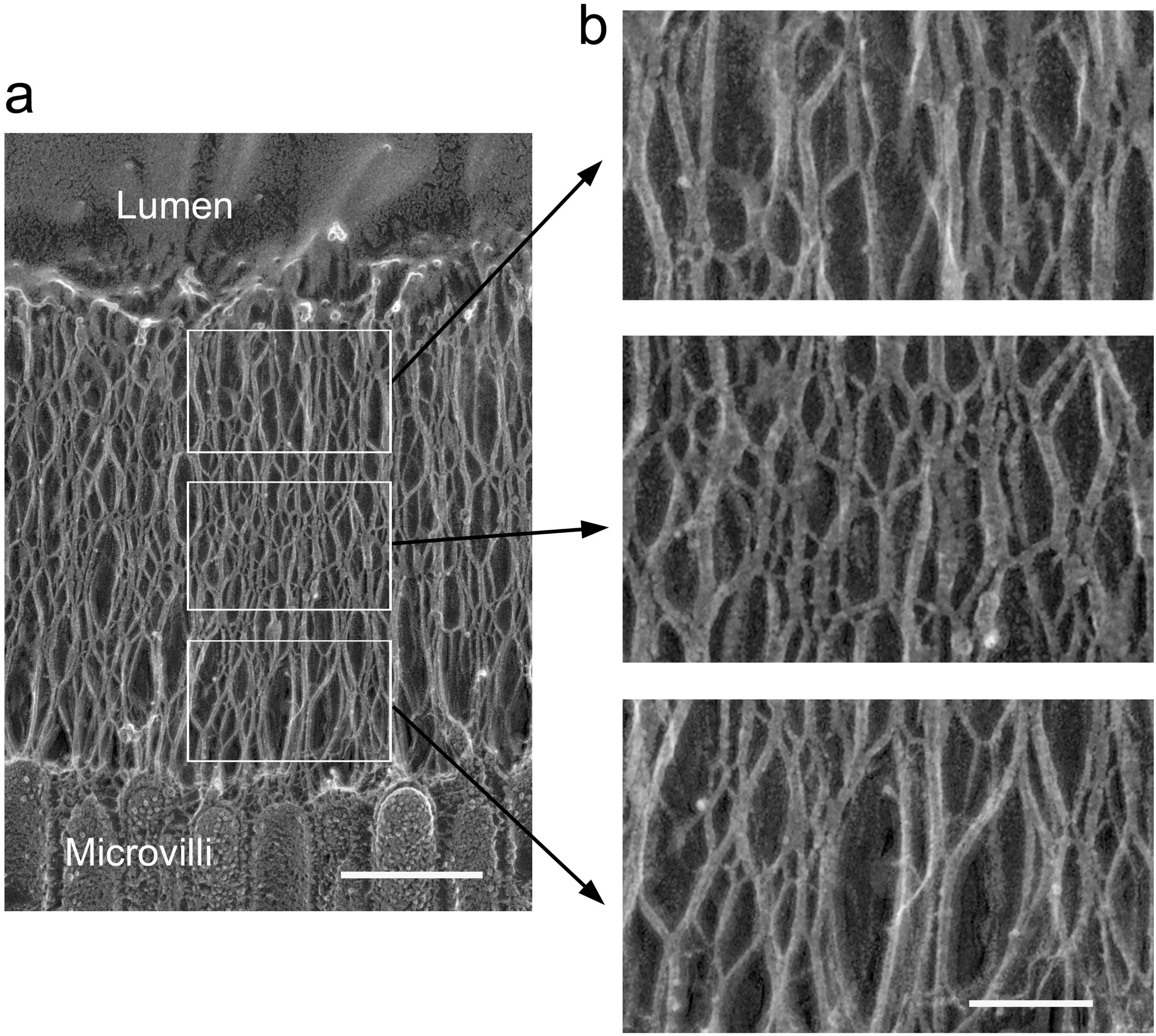

Supplementary Figure 2: The three-dimensional glycocalyx network shows stratified degrees of lateral interactions. The central zone of the glycocalyx network (**a**) has more extensive lateral interactions and a smaller meshwork relative to the region immediately flanking the luminal surface or emerging from the microvillar tips (**b**). Scale bars: **a** = 300 nm; **b** = 100 nm.

Supplementary figure 3

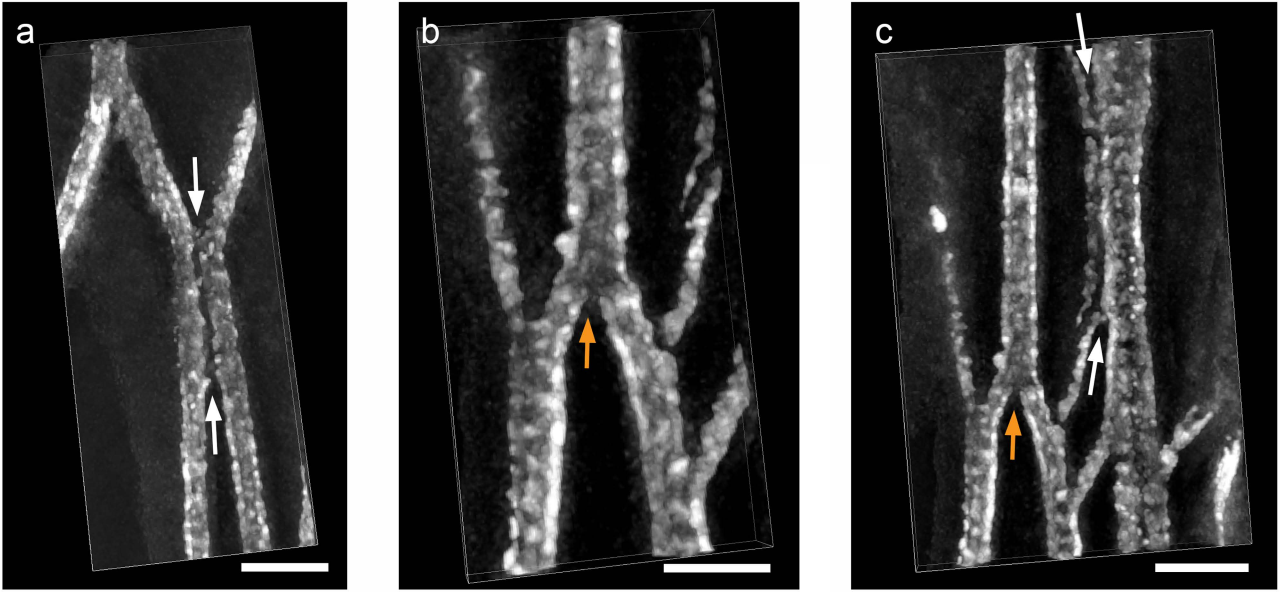

Supplementary Figure 3: Glycocalyx filaments make at least two types of lateral contacts. Three-dimensional rendering of tomographic reconstructions showing: (**a**) lateral contact with a gap (white arrows) between adjacent filaments, where both filaments remain as discrete units; **(b-c)** lateral contact (orange arrows) where no separation between the merging filaments could be resolved with the freeze-etching method, i.e., filaments appear to fuse into a thicker filament. Scale bars = 20 nm

Supplementary Figure 4

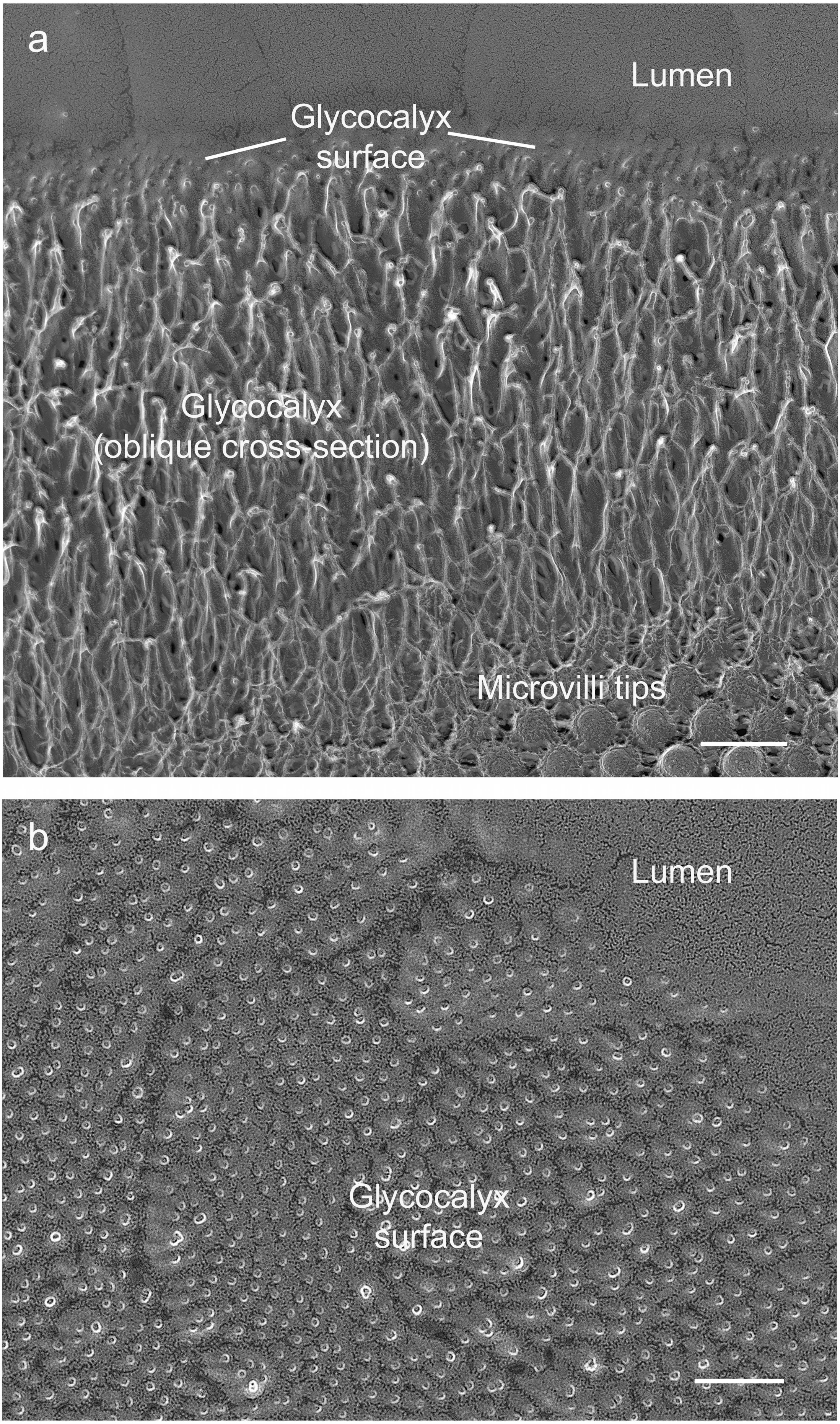

Supplementary Figure 4: Globular termini of glycocalyx filaments converge to form a single plane at the luminal interface. **(a)** An oblique cross-section view of the glycocalyx from termini to microvilli shows clearly the stratified organization of the microvillar tips, the glycocalyx layer of uniform thickness, the layer of globular heads of the filament termini forming the surface of the glycocalyx, and the intestinal lumen. **(b)** En face view of the luminal surface of the glycocalyx formed by the globular termini organized in a single uniform plane. Scale bars: **a** = 200 nm; **b**) 100 nm

Supplementary Figure 5

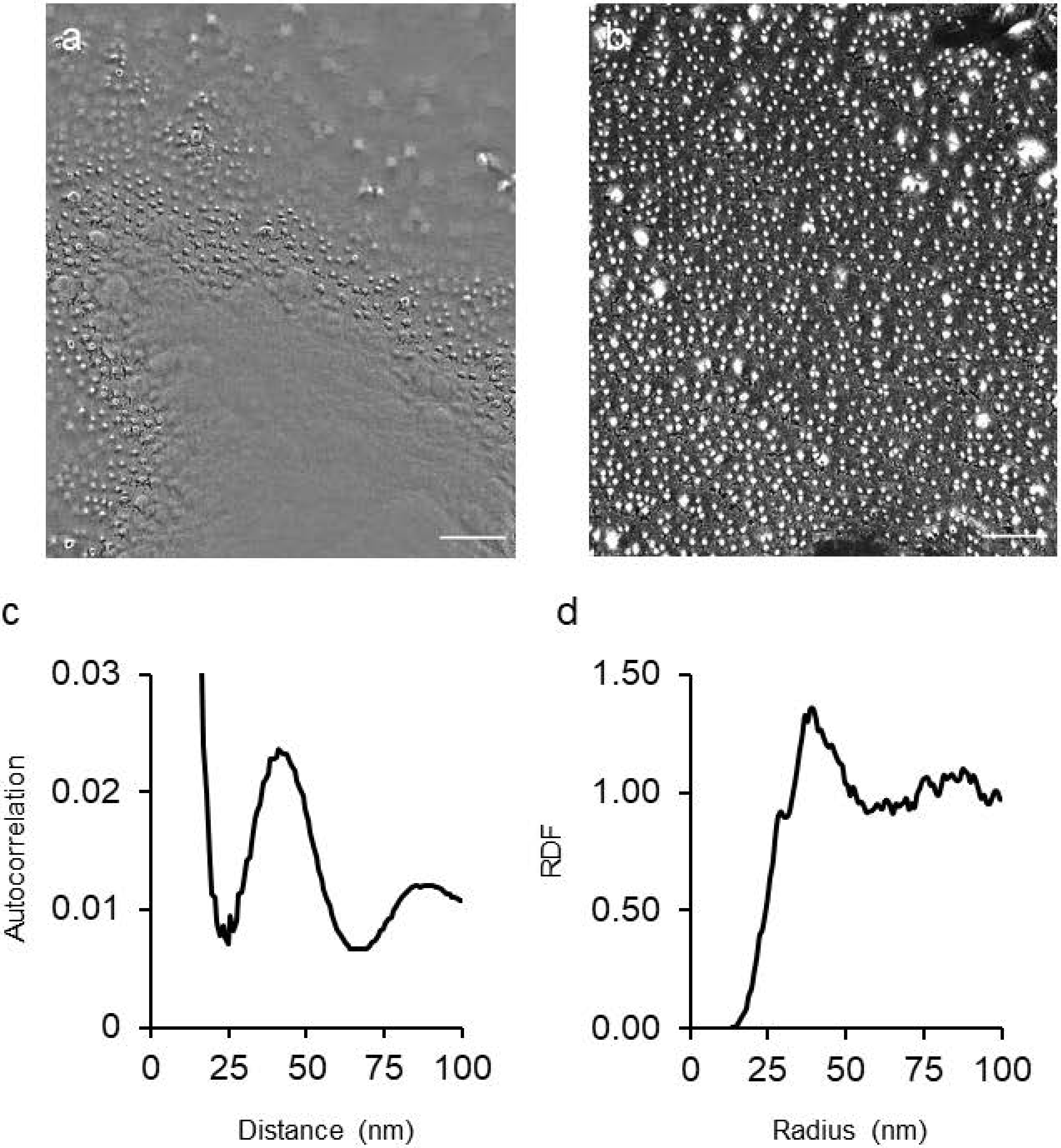

Supplementary Figure 5: Spatial analysis of glycocalyx termini. **(a)** a tomographic slice from the volume showing a portion of glycocalyx termini in focus and max intensity Z-projection of the tomographic volume highlighting all filament termini **(b)**. **(c)** Autocorrelation and radial distribution function **(d)** of the max intensity Z-projection. Scale bars: 200 nm

Supplementary Figure 6

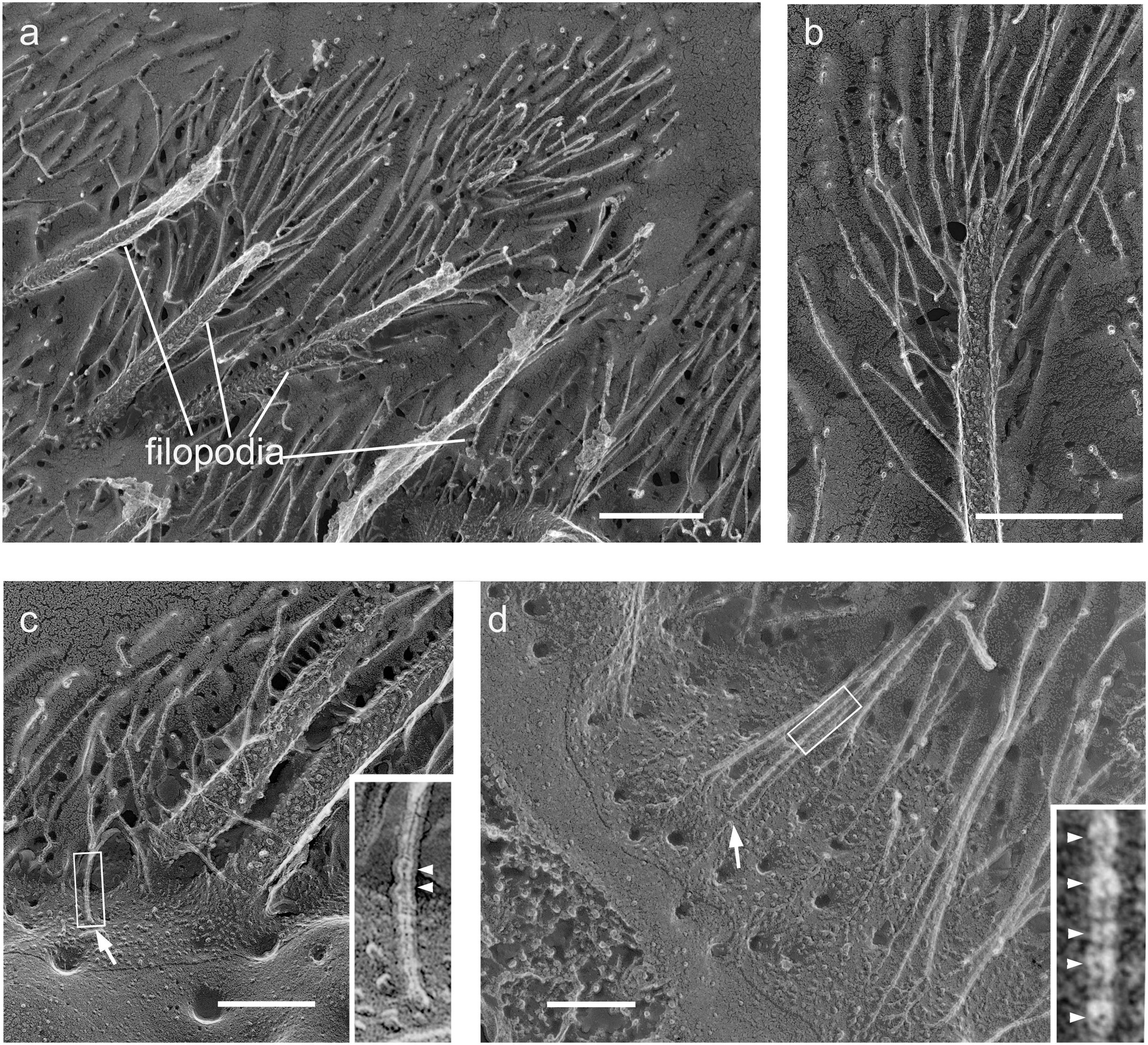

Supplementary Figure 6: The organization of the mesothelial glycocalyx differs from epithelial glycocalyx. **(a – d)** Freeze-etching of rat mesothelium surface. Glycocalyx filaments emerge from along the mesothelial filopodia and not just from the distal ends as with the case of intestinal glycocalyx. Filaments also emerge from the plasma membrane surface between numerous caveola (**c and d**, arrows). Minimal, if any, lateral interactions are observed between these glycocalyx filaments. Insets, enlargements of the rectangular areas showing that mesothelial glycocalyx filaments have a quasi-periodic substructure (arrowheads). Scale bars: **a, b** = 500 nm; **c, d** = 200nm
