# Supplementary material for: Nanoarchitecture and dynamics of the mouse enteric glycocalyx examined by freeze-etching electron tomography and intravital microscopy
Source: Commun Biol. 2020 Jan 7;3:5. doi: 10.1038/s42003-019-0735-5 (PMC6946683; doi:10.1038/s42003-019-0735-5)
Supplement: Supplementary file 2 — Description of additional supplementary files [file 42003_2019_735_MOESM2_ESM.pdf]

Supplementary Movie 1: Intravital microscopy of the glycocalyx in the intestinal lumen of an anesthetized mouse. The fluorescence signal is the result of 3 kDa dextran conjugated with fluorescein that was injected directly into the lumen before imaging. The fluid movement between intestinal villi is caused by peristaltic movement of the intestine and by cyclic breathing movements. Erythrocytes (visualized as dark objects excluding fluorescence), originating from the surgical disruption of blood vessels, flood the lumen. Erythrocytes consistently maintain a distance from the microvillar surface, even when squeezed between villi.

Supplementary Data 1

Source data for Figure 2c, measuring the maximum distance between filaments outlining open spaces within the glycocalyx meshwork.

Supplementary Data 2

Source data for Figure 2h, measuring the width of glycocalyx filaments by measuring the width of the impression the biological filament left on the replica.

Supplementary Data 3

Source data for Figure 3i, measuring nearest neighbor distance between glycocalyx filament termini in 3D from a large tomographic volume.

Supplementary Data 4

Source data for Figure 4b, showing the location of low and high molecular weight fluorescent dextrans.

Supplementary Data 5

Source data for Supplementary Figure 5c, output from FIJI autocorrelation plug-in.

Supplementary Data 6

Source data for Supplementary Figure 5d, output from FIJI radial distribution function plug-in.
