# Supplementary material for: Nanoarchitecture and dynamics of the mouse enteric glycocalyx examined by freeze-etching electron tomography and intravital microscopy
Source: Commun Biol. 2020 Jan 7;3:5. doi: 10.1038/s42003-019-0735-5 (PMC6946683; doi:10.1038/s42003-019-0735-5)
Supplement: Supplementary file 10 — Reporting Summary [file 42003_2019_735_MOESM10_ESM.pdf]

nature research

Corresponding author(s): Bechara Kachar
Last updated by author(s): Nov 13, 2019

Reporting Summary

Nature Research wishes to improve the reproducibility of the work that we publish. This form provides structure for consistency and transparency in reporting. For further information on Nature Research policies, see Authors & Referees and the Editorial Policy Checklist.

Statistics

For all statistical analyses, confirm that the following items are present in the figure legend, table legend, main text, or Methods section.

n/a	Confirmed	
☐	☒	The exact sample size (*n*) for each experimental group/condition, given as a discrete number and unit of measurement
☐	☒	A statement on whether measurements were taken from distinct samples or whether the same sample was measured repeatedly
☒	☐	The statistical test(s) used AND whether they are one- or two-sided<br>*Only common tests should be described solely by name; describe more complex techniques in the Methods section.*
☒	☐	A description of all covariates tested
☒	☐	A description of any assumptions or corrections, such as tests of normality and adjustment for multiple comparisons
☒	☐	A full description of the statistical parameters including central tendency (e.g. means) or other basic estimates (e.g. regression coefficient) AND variation (e.g. standard deviation) or associated estimates of uncertainty (e.g. confidence intervals)
☒	☐	For null hypothesis testing, the test statistic (e.g. *F*, *t*, *r*) with confidence intervals, effect sizes, degrees of freedom and *P* value noted<br>*Give P values as exact values whenever suitable.*
☒	☐	For Bayesian analysis, information on the choice of priors and Markov chain Monte Carlo settings
☒	☐	For hierarchical and complex designs, identification of the appropriate level for tests and full reporting of outcomes
☒	☐	Estimates of effect sizes (e.g. Cohen's *d*, Pearson's *r*), indicating how they were calculated

Our web collection on statistics for biologists contains articles on many of the points above.

Software and code

Policy information about availability of computer code

Data collection	Digital Micrograph (Gatan); SerialEM (referenced); Nikon Elements (Nikon)
Data analysis	MATLAB (MathWorks, version 9.4.0.813654); Amira (ThermoFisher); Nikon Elements (Nikon); FIJI (referenced); Digital Micrograph (Gatan)

For manuscripts utilizing custom algorithms or software that are central to the research but not yet described in published literature, software must be made available to editors/reviewers. We strongly encourage code deposition in a community repository (e.g. GitHub). See the Nature Research guidelines for submitting code & software for further information.

Data

Policy information about availability of data

All manuscripts must include a data availability statement. This statement should provide the following information, where applicable:

- Accession codes, unique identifiers, or web links for publicly available datasets
- A list of figures that have associated raw data
- A description of any restrictions on data availability

All data supporting the findings of this study are available from the corresponding author upon reasonable request.

Field-specific reporting

Please select the one below that is the best fit for your research. If you are not sure, read the appropriate sections before making your selection.

☒ Life sciences ☐ Behavioural & social sciences ☐ Ecological, evolutionary & environmental sciences

Life sciences study design

All studies must disclose on these points even when the disclosure is negative.

Sample size	Freeze-fracture and freeze-etching produce semi-random views of biological surfaces. We produced at least two replicas from each mouse's small intestine and we examined the intestinal epithelium of at least five mice. Individual replica was exhaustively examined for all areas of interest.<br><br>For immunolabelling, we examined at least two sets (4 sections in each set) of mouse small intestine cryo-section.
Data exclusions	No data was excluded.
Replication	Freeze-etching replicas and immunolabelling were performed at least twice per tissue per animal.
Randomization	There was no randomization.
Blinding	There was no blinding.

Reporting for specific materials, systems and methods

We require information from authors about some types of materials, experimental systems and methods used in many studies. Here, indicate whether each material, system or method listed is relevant to your study. If you are not sure if a list item applies to your research, read the appropriate section before selecting a response.

Materials & experimental systems

n/a	Involved in the study	
☐	☒	Antibodies
☒	☐	Eukaryotic cell lines
☒	☐	Palaeontology
☐	☒	Animals and other organisms
☒	☐	Human research participants
☒	☐	Clinical data

Methods

n/a	Involved in the study	
☒	☐	ChIP-seq
☒	☐	Flow cytometry
☒	☐	MRI-based neuroimaging

Antibodies

Antibodies used	Anti-MUC17, rabbit polyclonal, ab122184 (abcam)
Validation	Immunohistochemistry of anti-MUC17 has been validated by the manufacturer (abcam) on Paraffin-embedded human small intestine tissue. This antibody has previous been described and validated in Yang CW et al. Genetic variations of MUC17 are associated with endometriosis development and related infertility. BMC Med Genet 16:60 (2015).

Animals and other organisms

Policy information about studies involving animals; ARRIVE guidelines recommended for reporting animal research

Laboratory animals	Mouse strains: C57BL/6; mT/mGFP ; Rat strain: Sprague Dawley
Wild animals	N/A
Field-collected samples	N/A
Ethics oversight	NIDCD ACUC, protocol #1215; NCI ACUC, protocol #LCMB-031

Note that full information on the approval of the study protocol must also be provided in the manuscript.
